# Outcomes following oropharyngeal squamous cell carcinoma resection and bilateral neck dissection with or without contralateral postoperative radiotherapy of the pathologically node-negative neck

**DOI:** 10.1007/s00405-023-07972-4

**Published:** 2023-05-03

**Authors:** Florian Jansen, Christian Stephan Betz, Matthias Hans Belau, Gesa Matnjani, Till Sebastian Clauditz, Sebastian Dwertmann-Rico, Katharina Stölzel, Nikolaus Möckelmann, Arne Böttcher

**Affiliations:** 1 Department of Otorhinolaryngology, Head and Neuro Center, University Medical Center Hamburg-Eppendorf, Martinistrasse 52, 20246 Hamburg, Germany; 2 Institute of Medical Biometry and Epidemiology, University Medical Center Hamburg-Eppendorf, Hamburg, Germany; 3 Department of Radiotherapy, University Medical Center Hamburg-Eppendorf, Hamburg, Germany; 4 Institue of Pathology, University Medical Center Hamburg-Eppendorf, Hamburg, Germany; 5 Department of Otorhinolaryngology, Kath. Marienkrankenhaus GmbH, Hamburg, Germany

**Keywords:** Adjuvant treatment, Oropharyngeal cancer, Contralateral neck, Neck irradiation, HPV

## Abstract

**Purpose:**

There are no consensus guidelines regarding the postoperative treatment of the contralateral pathologically node-negative neck in oropharyngeal squamous cell carcinoma. This study aimed to determine if omission of postoperative irradiation of the contralateral pathologically node-negative neck affects oncological outcomes.

**Methods:**

We retrospectively identified 84 patients with primary surgical treatment including bilateral neck dissection and postoperative (chemo-)radiotherapy (PO(C)RT). Survival was analyzed using the log-rank test and the Kaplan–Meier method.

**Results:**

Patients showed no decrease in tumor-free, cause-specific (CSS), or overall survival (OS) when PO(C)RT of the contralateral pathologically node-negative neck was omitted. Increased OS was found in patients with unilateral PO(C)RT and especially an increased OS and CSS was found in unilateral PO(C)RT and in tumors arising from lymphoepithelial tissue.

**Conclusions:**

Omitting the contralateral pathologically node-negative neck appears to be safe in terms of survival and our retrospective study advocates further prospective randomized control de-escalation trials.

## Introduction

Squamous cell carcinoma of the head and neck (HNSCC) is the sixth most common malignant tumor entity worldwide with an incidence of 890,000 new cases and 450,000 deaths in 2018 [1]. Prolonged exposure to tobacco, tobacco-related products, and alcohol is known to increase the risk of developing HNSCC [2]. Squamous cell carcinoma of the oropharynx (OPSCC) is one of the various tumor entities in the head and neck region with a steadily increasing incidence of more than 400,000 cases per year worldwide [3]. In addition, the identification of exposure to high-risk oncogenic human papillomavirus (HPV) was observed to be a risk factor for developing OPSCCs, particularly in young adult males with no smoking history [4, 5].

A review of the international study landscape showed that the proportion of HPV-associated oropharyngeal carcinomas increased from 40.5% in studies conducted before 2000 to 72.2% in studies conducted after 2005, underscoring the extraordinary importance of this tumor entity [6]. Furthermore, it was shown that a positive HPV status in OPSCC was associated with a significantly improved survival [overall as well as tumor-free survival (OS/TFS)], as this population has fewer comorbidities and responds better to curative radiation and cisplatin [7]. This improved response is presumably due to the fact that HPV-mediated tumorigenesis and viral oncoproteins alter distinctive DNA double-strand break repair mechanisms and thereby the response to DNA damage by radiotherapy (RT) improves antitumor immunity, results in an increased rate of apoptosis, and gives rise to cell cycle alteration [8, 9]. It was also shown that HPV status was a more important factor in 5-year survival than T status or nodal status, which was ultimately reflected in a revised TNM Classification of Malignant Tumors (TNM), 7th and 8th editions [10–12].

OPSCCs, especially in locally advanced stages, often require multimodal treatment which includes tumor resection followed by postoperative radiotherapy (PORT) or chemoradiation (POCRT) in advanced stages, or organ preservation using definitive chemoradiation (CRT) [13]. Regarding the surgical management of neck lymph nodes, the National Comprehensive Cancer Network (NCCN) as well as Association of Scientific Medical Society in Germany (AWMF) provides treatment recommendations. Here, a distinction can be made between radical, modified radical and selective neck dissection, with less radical operations sparing the sternocleidomastoid muscle, the jugular vein, the spinal accessory nerve or selective lymph node levels, and have been developed based on the common pathways for regional head and neck metastases to remove all possibly involved lymph nodes or non-lymphatic structures [14, 15]. In case of clinical N0 status and no further indications for a possible bilateral involvement due to the localization and characteristics of the primary tumor, a selective neck dissection of the neck lymph node levels II–IV and V when appropriate of the ipsilateral side should be performed. If the clinical status is N+, at least a modified radical neck dissection of the involved side of the neck and at least a selective neck dissection on the contralateral side should be performed. If there is clinical evidence of bilateral involvement, at least a modified radical neck dissection should be performed on both sides [16–18]. POCRT is recommended in patients with high-risk pathologic features, such as extranodal extension (ENE) or positive margins. PORT alone is recommended in patients with intermediate-risk pathologic features, such as positive neck nodes, advanced T status (T3 or T4), or several minor risk criteria, such as perineural infiltration [19].

At present, intensity-modulated radiotherapy (IMRT), a computer-assisted irradiation technique with a dose of 60–66 Gy to the surgical bed and affected lymph nodes, is regularly used for PO(C)RT [20]. The exact dose of RT to the target clinical volume is determined based on the aforementioned pathologic risk criteria and is administered in 30–33 fractions, with a dose of 60 Gy given when intermediate-risk criteria are present and boosted to 66 Gy when high-risk criteria are met. Moreover, earlier studies have recommended that clinically, radiologically, and pathologically uninvolved lymph node regions should also be irradiated postoperatively with a dose of 52–54 Gy in 30–33 fractions as additional clinical target volumes to eradicate occult metastases. This approach is also dependent on whether intermediate- or high-risk criteria are present [21, 22]. Furthermore, because of different lymphatic drainage and increased likelihood of midline proximity, which is associated with an increased probability of occult metastases of the contralateral side of the neck, bilateral radiation may be indicated for tumors of the base of the tongue compared with tumors of the tonsil [23, 24]. However, neither the recent guidelines of the NCCN nor those of the Association of the Scientific Medical Societies in Germany formulate precise instructions on how the lymphatic drainage pathways should be irradiated, especially für pathologically negative pathways, and do not differentiate between p16-negative and p16-positive OPSCC [16, 17, 19]. Thus, patients with pathologically non-involved cervical lymph node regions, or even entire non-involved neck sides, experience different RT regimens postoperatively, depending on the tumor board discussion or the treating physician.

To date, there are no accepted guidelines for PO(C)RT of the pathologically node-negative contralateral neck in patients with OPSCC. This study aimed to determine if the omission of postoperative radiotherapy of the contralateral pathologically node-negative neck affects oncologic outcomes in a distinct patient cohort.

## Materials and methods

### Patients

We performed a retrospective patient chart review of women and men with OPSCC diagnosed between January 2010 and January 2020 with primary surgical treatment, including bilateral neck dissection based on clinical evidence for either cervical lymph node involvement (cN+) or evidence based on the location of the primary tumor for possible bilateral involvement according to NCCN and AWMF recommendations, followed by PO(C)RT. Regarding unilateral or bilateral irradiation of the neck, the decision was based on the tumor board discussion and made by the treating radiation therapists, mainly depending on the midline proximity and size of the tumor, the depth of infiltration into the base of the tongue, and number of pathologically involved cervical lymph nodes. The exclusion criteria included previous head and neck malignancies and disease progression during treatment. Using trained coordinators, data were obtained from a database management system using the Gießen Tumor Documentation System (GTDS; https://www.med.uni-giessen.de/akkk/gtds/), which thoroughly documents patients’ features using the original pathology report. In addition, a review of patients’ digital records was conducted using our local documentation systems myMedis KIS (Getinge) and Soarian^®^ Clinicals (Cerner). Cases were identified using the German modification of the International Classification of Diseases (ICD-10-GM) for Oncology topography codes C05.1–9, C09.-, C10.-, and C01.-. From an initial cohort of 347 patients, those without information on tumor stage, tumor region, patient characteristics, treatment protocol, relevant data regarding p16 status or margin status were excluded from the analyses (*n* = 231). Furthermore, we excluded patients with unknown lymph node status (*n* = 17), pathologically bilateral positive lymph nodes (*n* = 12) and involved margins (*n* = 3). There were 84 patients with ipsilateral pathologically positive lymph nodes and uni- or bilateral PO(C)RT in the final analytical sample and each patient1 was directly treated or examined during a follow-up routine by at least one of the authors.

### Endpoints

The primary endpoint was tumor-free survival (TFS), defined as duration from initial diagnosis to the date of tumor recurrence, nodal relapse, or distant metastasis occurrence. Secondary endpoints were cause-specific-survival (CSS), defined as duration from initial diagnosis to the date of death due to the diagnosed disease and overall survival (OS), defined as duration from initial diagnosis to the to the date of death due to any cause.

### Statistical analysis

Survival curves were generated using the Kaplan–Meier method for OS, TFS and CSS, using the log-rank test to assess differences in survival among various subgroups. Cases presenting a missing value for at least one of the modeling variables were excluded from the analyses (listwise deletion). Statistical significance was set at *p* < 0.05. All data analyses were performed using STATA MP, version 17 (Stata Corp LLC, College Station, TX, USA).

## Results

### Sample characteristics

Among the 84 included patients, irradiation was performed with IMRT with a dose of 60–66 Gy for the surgical bed and the region of affected lymph nodes with 30–33 fractions (once per day, five fractions per week), depending on pathologic risk factors. Uninvolved lymph node regions were irradiated with a dose of 50–54 Gy in 30–33 fractions. The mean follow-up period was 36.2 months (SD 27.6 months). The baseline characteristics and treatment data are shown in Table 1.

**Table 1:** Sample characteristics

	*n*	%
Age at diagnosis		
Mean (SD)	63.5 (9.1)	
Sex		
Male	58	69.1
Female	26	30.9
Tumor stage (AJCC8)		
I	55	65.5
II	16	19.1
III	5	5.9
IV	8	9.5
Tumor region		
BOT	21	25.0
Tonsil	56	66.7
Other	7	8.3
p16 status (IHC)		
Negative	10	11.9
Positive	74	88.1
HPV–DNA status		
Negative	16	20.3
Positive	63	79.7
*pN-stage (AJCC8)*		
p16 negative		
0	0	0.0
1	3	30.0
2	0	0.0
2a	1	10.0
2b	5	50.0
2c	0	0.0
3a	0	0.0
3b	1	10.0
p16 positive		
0	0	0.0
1	59	79.7
2	15	20.3
Irradiation site		
Unilateral	41	48.8
Bilateral	43	51.2
Extranodal extension		
Negative	43	51.2
Positive	39	46.4
Missing values	2	2.4
Margin status		
Clear	66	78.6
Close	18	21.4
Chemoradiation		
No	59	70.2
Yes	25	29.8

Other regions: palatopharyngeal arch, posterior pharyngeal wall, lateral pharyngeal wall, uvula

*n* quantity, *%* proportion, *SD* standard deviation, *BOT* base of tongue, *DNA* deoxyribonucleic acid, *HPV* human papilloma virus, *IHC* immunohistochemistry, *PCR* polymerase chain reaction

### Survival analysis

#### TFS of patients with unilateral versus bilateral irradiation

**Fig. 1:**
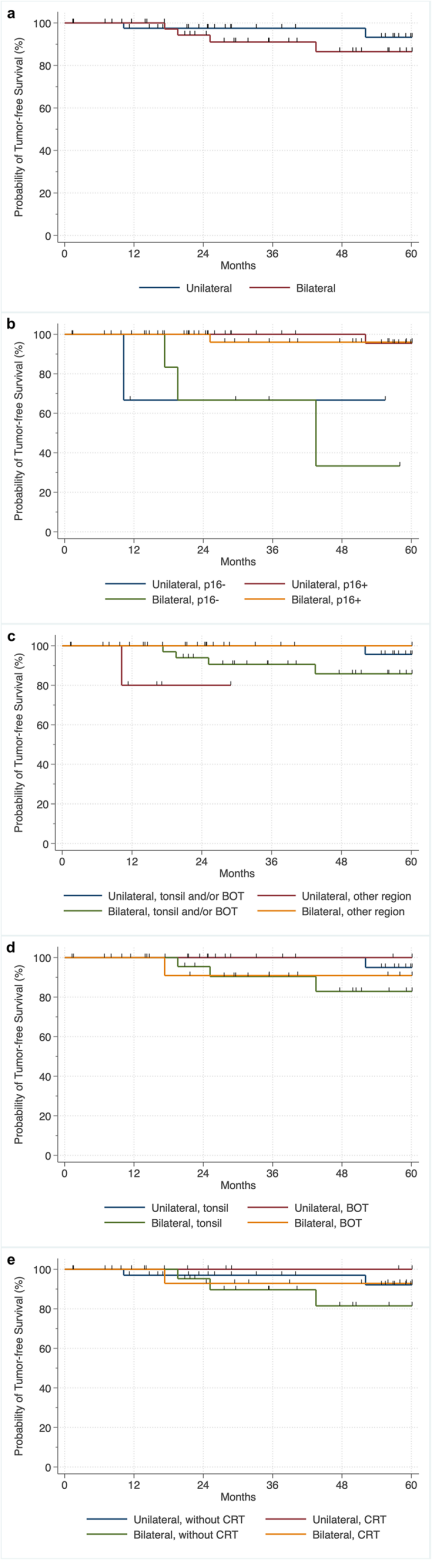
Kaplan–Meier plots of tumor-free survival showing **a** no survival difference when comparing unilateral-to-bilateral postoperative [chemo-)radiotherapy (PO(C)RT) (log-rank (global):* p* = 0.106], **b** no survival difference when comparing unilateral to bilateral PO(C)RT stratified by p16 status [log-rank (global): *p* < 0.772; log-rank (stratified): p16-positive: *p* = 0.843; p16-negative: *p* = 0.829], **c** no survival difference when comparing unilateral to bilateral (PO(C)RT stratified by tonsil and/or BOT versus other regions [log-rank (global): *p* = 0.160; log-rank (stratified): tonsil and/or BOT: *p* = 0.092; other regions: *p* = 0.925], **d** no survival difference when comparing unilateral to bilateral PO(C)RT stratified by tonsil versus BOT [log-rank (global): *p* = 0.072; log-rank (stratified): tonsil: *p* = 0.128; BOT: *p* = 0.339], and **e** no survival difference when comparing unilateral to bilateral PORT stratified whether or not CRT was performed [log-rank (global): *p* = 0.085; log-rank (stratified): without CRT: *p* = 0.161; CRT: *p* = 0.307]. Other regions: palatopharyngeal arch, posterior pharyngeal wall, lateral pharyngeal wall, uvula. *BOT* base of tongue, *CRT* chemoradiotherapy

There were no differences in TFS between patients who had been irradiated unilaterally or bilaterally, with 5-year survival rates of 93.3% for unilateral irradiation and 86.9% for bilateral irradiation (*p* = 0.106) (Fig. 1a). In addition, stratified analyses by p16 status showed no correlations in TFS regardless of whether unilateral or bilateral irradiation was performed, with 5-year survival rates of 95.5% for unilateral irradiation and 96.2% for bilateral irradiation in p16 positivity, and 5-year survival rates of 66.7% for unilateral irradiation and 16.7% for bilateral irradiation in p16 negativity (global: *p* < 0.772; p16-positive: *p* = 0.843; p16-negative: *p* = 0.829) (Fig. 1b).

Stratified analyses of TFS by region (tonsil and/or BOT versus palatopharyngeal arch/posterior pharyngeal wall/lateral pharyngeal wall/uvula, also described as "other") showed no correlations in TFS regardless of whether unilateral or bilateral irradiation was performed, with 5-year survival rates of 95.7% for unilateral irradiation and 86.2% for bilateral irradiation in tumors arising from the tonsil and/or BOT, and a 5-year survival rate of 100% for bilateral irradiation in tumors arising from a region declared as "other" (global: *p* = 0.160; tonsil and/or BOT region: *p* = 0.092; other region: *p* = 0.925). A statement on the 5-year survival rate was not possible in patients with unilateral irradiation and with tumors arising from the region declared as "other" because of censoring (Fig. 1c).

Stratified analyses by region (tonsil versus BOT) showed no correlations in TFS regardless of whether unilateral or bilateral irradiation was performed, with 5-year survival rates of 95% for unilateral irradiation and 83.1% for bilateral irradiation in tumor arising from the palatine tonsil, and 5-year survival rates of 100.% for unilateral irradiation and 91% for bilateral irradiation in tumor arising from the BOT (global: *p* = 0.072; tonsil: *p* = 0.128; BOT: *p* = 0.339) (Fig. 1d).

Stratified analyses of TFS according whether or not CRT was performed showed no correlation in TFS regardless of whether unilateral or bilateral irradiation was performed, with 5-year survival rates of 92.1% for unilateral irradiation and 81.1% for bilateral irradiation without CRT, and 5-year survival rates of 100% for unilateral irradiation and 94.2% for bilateral irradiation and CRT (global: *p* = 0.085; without CRT: *p* = 0.161; CRT: *p* = 0.307) (Fig. 1e).

#### CSS of patients with unilateral versus bilateral irradiation

**Fig. 2:**
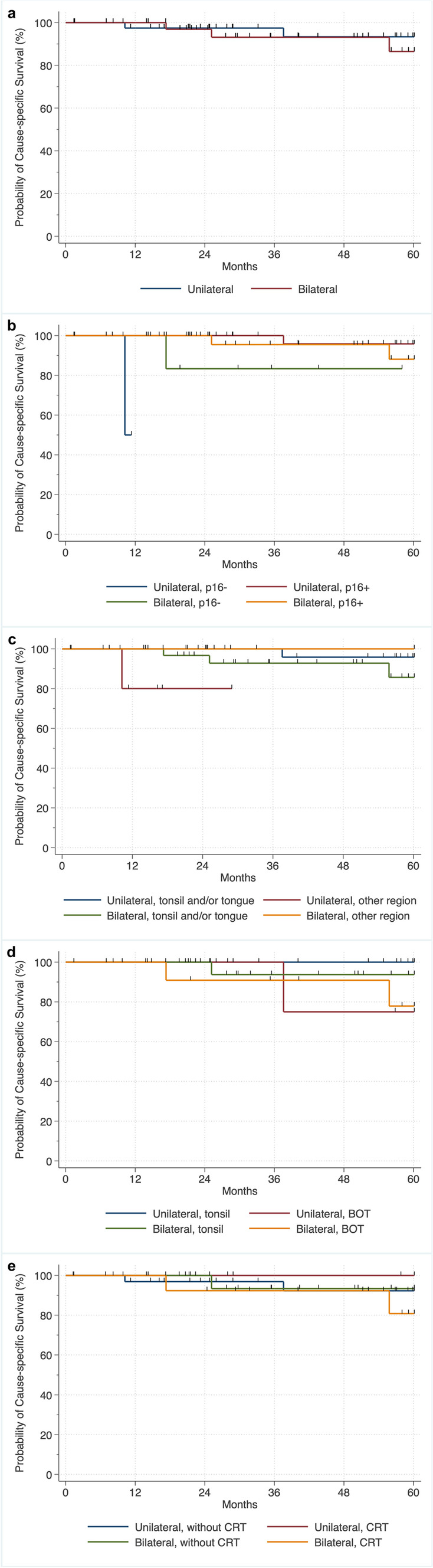
Kaplan–Meier plots of cause-specific survival showing **a** no survival difference when comparing unilateral to bilateral postoperative [chemo-)radiotherapy (PO(C)RT) (log-rank (global): *p* = 0.172], **b** no survival difference when comparing unilateral to bilateral PO(C)RT stratified by p16 status [log-rank (global): *p* < 0.310; log-rank (stratified): p16-positive: *p* = 0.068; p16-negative: *p* = 0.601], **c** survival difference when comparing unilateral to bilateral (PO(C)RT stratified by region with improved CSS in patients with unilateral PO(C)RT with tumors declared as tonsil and/or BOT [log-rank (global): *p* = 0.062; log-rank (stratified): tonsil and/or BOT: *p* = 0.029; other regions: *p* = 0.786], **d** no survival difference when comparing unilateral to bilateral (PO(C)RT stratified by tonsil versus BOT [log-rank (global): *p* = 0.062; log-rank (stratified): tonsil: *p* = 0.053; BOT: *p* = 0.462], and **e** no survival difference when comparing unilateral to bilateral PORT stratified whether or not CRT was performed [log-rank (global): *p* = 0.056; log-rank (stratified): without CRT: *p* = 0.186; CRT: *p* = 0.152]. Other regions: palatopharyngeal arch, posterior pharyngeal wall, lateral pharyngeal wall, uvula. *BOT* base of tongue, *CRT* chemoradiotherapy

There were no differences in CSS in patients irradiated unilaterally or bilaterally with 5-year survival rates of 93.4% for unilateral irradiation and 87.6% for bilateral irradiation (*p* = 0.172) (Fig. 2a). Furthermore, stratified analyses by p16 status showed no differences in CSS regardless of whether unilateral or bilateral irradiation was performed, with 5-year survival rates of 95.9% for unilateral irradiation and 89.5% for bilateral irradiation in p16 positivity and a 5-year survival rate of 83.4% for bilateral irradiation in p16 negativity (global: *p* < 0.310; p16-positive: *p* = 0.068; p16-negative: *p* = 0.601). A statement on the 5-year survival rate was not possible in patients with unilateral irradiation and p16 negativity due to censoring (Fig. 2b).

Stratified analyses of CSS by region (tonsil and/or BOT versus “other”) showed improved CSS in patients with unilateral irradiation with tumors arising in a region declared as tonsil and/or BOT compared to patients with bilateral irradiation and tumors in the same region throughout the observation period of up to 60 months, with 5-year survival rates of 95.9% for unilateral irradiation and 86.8% for bilateral irradiation (global: *p* = 0.062; tonsil and/or BOT: *p* = 0.029). In addition, no correlations were present in CSS in patients with tumors arising from a region declared as “other”, regardless of whether unilateral or bilateral irradiation was performed, with a 5-year survival rate of for 100% for bilateral irradiation (other regions: *p* = 0.786) (Fig. 2c). A statement about the 5-year survival rate was not possible in patients with unilateral irradiation and with tumors arising fr2om the region declared as "other" due to censoring (Fig. 2c).

Stratified analyses by region (tonsil versus BOT) and by irradiation side showed no differences in CSS in patients regardless of whether unilateral or bilateral irradiation was performed, with 5-year survival rates of 100% for unilateral irradiation and 93.8% for bilateral irradiation in tumors arising from the tonsil, and a 5-year survival rate of 75% for unilateral irradiation and 76.7% for bilateral irradiation in tumors arising from a region declared as BOT (global: *p* = 0.062; tonsil and/or BOT region: *p* = 0.053; other region: *p* = 0.462) (Fig. 2d).

Stratified analyses of CCS according whether or not CRT was performed showed no correlation in CCS regardless of whether unilateral or bilateral irradiation was performed, with 5-year survival rates of 92.3% for unilateral irradiation and 93.4% for bilateral irradiation without CRT, and 5-year survival rates of 100% for unilateral irradiation and 82.7% for bilateral irradiation and CRT (global: *p* = 0.056; without CRT: *p* = 0.186; CRT: *p* = 0.152) (Fig. 2e).

#### OS of patients with unilateral versus bilateral irradiation

**Fig. 3:**
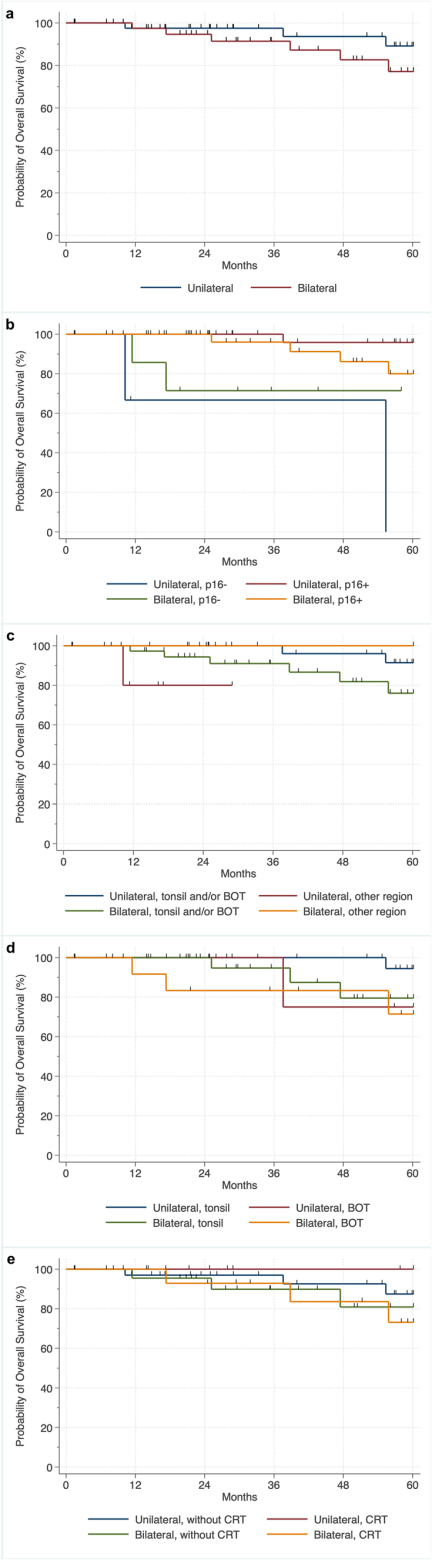
Kaplan–Meier plots of overall survival (OS) showing **a** survival difference when comparing unilateral to bilateral postoperative [chemo-)radiotherapy (PO(C)RT) with improved survival in patients with unilateral PO(C)RT (log-rank (global): *p* = 0.043], **b** no survival difference when comparing unilateral to bilateral PO(C)RT stratified by p16 status [log-rank (global): *p* < 0.310; log-rank (stratified): p16-positive:* p* = 0.068; p16-negative: *p* = 0.601], **c** survival difference when comparing unilateral to bilateral PO(C)RT stratified by region with improved OS in patients with unilateral PO(C)RT with tumors in a region declared as tonsils and/or BOT [log-rank (global): *p* = 0.062; log-rank (stratified): tonsil and/or BOT: *p* = 0.029; other regions: *p* = 0.786], **d** no survival difference when comparing unilateral to bilateral (PO(C)RT stratified by tonsil versus BOT [log-rank (global): *p* = 0.098; log-rank (stratified): tonsil: *p* = 0.154; BOT: *p* = 0.386], and **e** no survival difference when comparing unilateral to bilateral PORT stratified whether or not CRT was performed [log-rank (global): *p* = 0.056; log-rank (stratified): without CRT: *p* = 0.186; CRT: *p* = 0.152]. Other regions: palatopharyngeal arch, posterior pharyngeal wall, lateral pharyngeal wall, uvula. *BOT* base of tongue, *CRT* chemoradiotherapy

Analyses by irradiation side showed differences in OS when comparing unilateral to bilateral PO(C)RT with improved survival in patients with unilateral PO(C)RT, with 5-year survival rates of 89.3% for unilateral irradiation and 77.1% for bilateral irradiation (*p* = 0.043) (Fig. 3a). In addition, stratified analyses by p16 status showed no correlation with OS in patients regardless of whether unilateral or bilateral irradiation was performed, with 5-year survival rates of 95.9% for unilateral irradiation and 80.3% for bilateral irradiation in the case of p16 positivity and 5-year survival rates of 69.1% for bilateral irradiation in the case of p16 negativity (global: *p* < 0.310; p16-positive:* p* = 0.068; p16-negative: *p* = 0.386). A statement about the 5-year survival rate was not possible in patients with unilateral irradiation and p16 negativity due to censoring (Fig. 3b).

Furthermore, stratified analyses by region (tonsil and/or BOT versus “other”) and by irradiation side showed markedly improved OS in patients with unilateral irradiation with tumors in a region declared as tonsil and/or BOT compared to patients with bilateral irradiation and tumors in the same region throughout the observation period of up to 60 months, with 5-year survival rates of 91.7% for unilateral irradiation and 75.1% for bilateral irradiation (global: *p* = 0.062; tonsil and/or BOT region: *p* = 0.029). Moreover, no correlations were present in OS in patients with tumors arising from a region declared as "other", regardless of whether unilateral or bilateral irradiation was performed, with a 5-year survival rate of 100% for bilateral irradiation (other region: *p* = 0.786). A statement about the 5-year survival rate was not possible in patients with unilateral irradiation and with tumors arising from the region declared as "other" due to censoring (Fig. 3c).

Stratified analyses by region (tonsil versus BOT) showed no correlations in OS regardless of whether unilateral or bilateral irradiation was performed, with 5-year survival rates of 95% for unilateral irradiation and 79.4% for bilateral irradiation in tumor arising from the palatine tonsil, and 5-year survival rates of 75.% for unilateral irradiation and 69.5% for bilateral irradiation in tumor arising from the BOT (global: *p* = 0.098; tonsil: *p* = 0.154; BOT: *p* = 0.386) (Fig. 3d).

Stratified analyses of OS according whether or not CRT was performed showed no correlation in CCS regardless of whether unilateral or bilateral irradiation was performed, with 5-year survival rates of 87.6% for unilateral irradiation and 81.8% for bilateral irradiation without CRT, and 5-year survival rates of 100% for unilateral irradiation and 84.2% for bilateral irradiation and CRT (global: *p* = 0.056; without CRT: *p* = 0.186; CRT: *p* = 0.152) (Fig. 3e).

## Discussion

The main objective of this study was to investigate the possible impact of omission of PORT to the contralateral pathologically node-negative neck on disease control and patient survival, contributing to a higher risk of tumor recurrence or death. Over a maximum observation period of 60 months, there was no worsening of TFS, CSS, or OS in our patient cohort when only unilateral irradiation was performed, thus omitting the irradiation of the contralateral node-negative side of the neck. This is consistent with studies that have already investigated the reduction of radiation dose on patient survival, particularly in HPV-driven disease [25, 26]. Because patients with HPV-driven OPSCC have a lower risk of death and are younger, the side effects and long-term effects of CRT and RT may be more apparent here, including dermatitis, mucositis, and dysphagia as well as chronic xerostomia, which can have an extraordinary influence on quality of life [27, 28]. Nevertheless, the radiation dose to the neck organs, especially the salivary glands, oral cavity, mandible, laryngeal structures, esophagus, and brachial plexus, is considered to be a risk factor for side effects [29–31]. Consequently, a corresponding reduction of radiation dose can lead to improved quality of life with improved swallowing function and reduced xerostomia, regardless of whether HPV-driven disease is present.

While there are many studies investigating a reduction in radiation dose, few have studied whether unaffected cervical lymph nodes need to be irradiated, although studies have consistently confirmed that the lymph node metastasis rate of the contralateral neck is significantly lower than that of the ipsilateral neck [32]. For example, patients with OPSCC receiving bilateral PO(C)RT have already been shown to have safe omission of radiologically uninvolved contralateral retropharyngeal lymph nodes and cervical region II lymph nodes, leading to a reduced contralateral parotid dose [33]. In this regard, our data are consistent even show a proportionally slightly better OS in patients with unilateral irradiation as a secondary endpoint. In this regard, bilateral irradiation may lead to a poorer prognosis due to the increased occurrence of side effects and long-term damage, which may justify the comparatively poorer prognosis in this specific comparison. Although this correlation should not be overestimated in a retrospective analysis, it appears to support the statement that PORT of the pathologically node-negative neck side does not improve patient survival and could, therefore, besides a dose reduction, be part of a de-escalation strategy in future trials.

Regarding the positive and negative p16 status in our distinct cohort, there were no differences in terms of TFS, CSS, or OS, regardless of whether unilateral or bilateral PO(C)RT was performed. Thus, it appears in our cohort that a pathologically negative neck site, even in p16-negative tumors, does not require PORT from a patient survival perspective and does not affect the generally worse prognosis of these patients, which should definitely be verified by prospective controlled comparative studies. Similarly, in our cohort, the proven equal prognosis of unilateral and bilateral PORT in only unilateral pathologically positive cervical lymph nodes of p16-positive and p16-negative OPSSC could support a de-escalation of therapy in the sense of omitting the contralateral node-negative neck. This underscores the need not only for a differentiated consideration of p16-positive and p16-negative OPSSCs as distinct tumor entities, but also for more prospective randomized control studies to prove this hypothesis and eventually adapt the RT guidelines, especially with regard to p16-negative tumors, as previously, de-escalation has mainly been requested only for p16-positive tumors.

Stratified by region, our data showed increased CSS and OS in patients with unilateral PO(C)RT with tumors in a region declared as tonsil and/or BOT, and, therefore, mainly lymphoepithelial tissue, compared with bilateral PO(C)RT. When the tumor originated from the palatopharyngeal arch, the posterior and lateral pharyngeal walls, or the uvula, and was, therefore, predominantly non-lymphoepithelial tissue, no differences in survival were observed when unilateral and bilateral PO(C)RT were compared. Focusing on OPSCC and the distinction in terms of p16 status and HPV status, many studies have not considered the oropharyngeal subsites and few studies have followed up with histopathological investigations in this regard [34]. Moreover, histopathology showed that OPSCCs could be divided into lymphoepithelial and non-lymphoepithelial subcategories, which also led us to examine our patients with regard to the corresponding subregion. Here, HPV prevalence was shown to be higher in lymphoepithelial tissue with significant discordance of HPV DNA and p16 positivity compared to non-lymphoepithelial tissue [35]. Furthermore, patients with OPSCC arising from lymphoepithelial tissue (tonsils, BOT) had a better clinical outcome when their tumors were both HPV DNA- and p16-positive than when they were only p16-positive, whereas a similar difference was not observed in patients with tumors that were histomorphologically not arising from lymphoepithelial tissue [36]. In our cohort, unilateral irradiation of tumors of lymphoepithelial origin not only appears to be sufficient, but even outperforms the prognosis of bilateral irradiation. In line with this, another study reported that patients with HPV-positive lymphoepithelial tonsillar or BOT carcinoma were associated with a better clinical prognosis. However, in comparison, the authors found no difference in prognosis in patients with HPV-positive OPSCC starting from non-lymphoepithelial tissue [37].

In terms of survival and tumors of the tonsil and BOT, our study found no association between OS, CSS and TFS and unilateral or bilateral irradiation. This is particularly interesting, because tumors of the BOT may be more midline and thus could theoretically tempt the radiation therapist to perform bilateral irradiation. Nevertheless, the lack of correlation is consistent with the suggestion of Last et al., who suspects that patients do not benefit from contralateral irradiation when the neck is pathologically negative for tumors of the BOT [24]. Therefore, according to our data, it may be considered to include and investigate the omission of irradiation of the contralateral side in further prospective randomized comparative studies regarding patient survival in case of unilateral positive cervical lymph nodes. With regard to tumors of non-lymphoepithelial origin, no better prognosis can be observed than when only unilateral irradiation is used, even if there is a generally lower probability of HPV-driven disease. Thus, this appears to be a similar situation to patients with a proven negative p16 status, where, in the presence of a pathologically negative neck, bilateral irradiation does not affect survival in our cohort. Hence, it does not seem to make a difference whether irradiation is performed on both sides, further investigation in the form of prospective randomized comparative studies should, therefore, be considered here as well.

To investigate whether high-risk factors such as ENE affect survival in patients who have received guideline-guided POCRT, we divided the cohort into those with or without CRT. Here, there were no differences in TFS, CSS, or OS between uni- or bilateral PORT, regardless of whether or not CRT was performed. This could imply that in our retrospective analysis, patients with high-risk factors do not necessarily benefit from irradiation of the pathologically node-negative neck side and concurrent adjuvant chemotherapy. These findings may be in the same line as several clinical trials involving de-escalation of postoperative therapy in patients with HPV positive OPSCCs. For example, the MC1273 trial demonstrated comparable locoregional control to historical controls after reduced postoperative radiation dose and concurrent chemotherapy, even in patients with ENE [38]. Other studies such as the ECOG 3311 (NCT01898494), the Adjuvant De-escalation, Extracapsular Spread, p16 Positive, Transoral (ADEPT) study, the DART–HPV (De-Escalated Adjuvant Radiation Therapy for HPV-Associated Oropharyngeal Cancer) study (NCT02908477), or the single institution study at the University of Pennsylvania (NCT02159703) are investigating different postoperative deintensification strategies, such as a lower radiation dose, a shorter radiation regimen, or omitting radiation in certain regions such as the tumor bed, with some studies already having data to support deintensification [39–42]. In this regard, it should be noted that our cohort is also primarily comprised of patients with HPV-driven disease. It is important that de-intensification strategies are thoroughly investigated in prospective studies to best define the clinical parameters for treatment decisions. Accordingly, we are eagerly awaiting the results of this and other numerous ongoing clinical trials to best help this patient population and hope that our retrospective data can assist in the further development of hypotheses regarding this significant disease entity.

In conclusion, we believe that certain factors should be considered and investigated in further studies regarding omission of PORT on the contralateral pathologically node-negative side of the neck. In particular, unilateral irradiation should be evaluated when the tumor occurs in lymphoepithelial tissue, because TFS, CSS, and OS were not only equivalent to bilateral irradiation, but OS and CSS even exceeded that of bilateral irradiation. It should be emphasized in this context that this also applies to tumors of the BOT with a pathologically negative neck. In this regard, it should be said that prospective studies are currently already being conducted, especially dose reduction of RT and CRT in HPV positive OPSCCs, which show better functional as well as oncologic outcome and also advocate larger, multicentric control studies [43, 44].

Therefore, our study, along with others, support prospective randomized comparative studies to test our hypotheses and recommends actions to revise existing guidelines to better standardize and de-intensify radiotherapy in terms of omitting PORT of the contralateral pathologically node-negative neck, which could hereby be part of a de-escalation strategy in future trials to reduce side effects.

## Strength and limitations

Limitations include the purely retrospective evaluation of the cohort and inclusion of only operated patients with unilateral positive lymph nodes and that a majority of OPSCC originate from lymphoepithelial tissue and are p16-positive. This circumstance limits the statements on p16-negative tumors. In addition, regarding possible confounding variables, such as TNM stage, ENE, margin status or performance of CRT, it should be mentioned that a confounding effect cannot be excluded as the groups might differ regarding these factors. Furthermore, the limited sample size due to a large documentation failure, the low number of tumors in higher stages, as well as the partly enlarged confidence intervals, must be mentioned as possible limitations. In addition, no midline proximity of the tumors could be regularly traced in our data, which would be quite interesting for further investigations regarding bilateral irradiation. The strengths of this study are its cohort from a tertiary referral university hospital, and its restrictively condensed nature with a small corridor of clinical features.

## Data Availability

The data come from an internal clinic data depository, these were anonymized and can, therefore, not be traced, the raw data are available at any time on request.
